# E-cigarette use behaviors and device characteristics of daily exclusive e-cigarette users in Maryland: Implications for product toxicity

**DOI:** 10.18332/tid/128319

**Published:** 2020-11-10

**Authors:** Angela Aherrera, Atul Aravindakshan, Stephanie Jarmul, Pablo Olmedo, Rui Chen, Joanna E. Cohen, Ana Navas-Acien, Ana M. Rule

**Affiliations:** 1 Department of Environmental Health and Engineering, Johns Hopkins Bloomberg School of Public Health, Baltimore, United States; 2 Division of Pediatric Pulmonary Medicine, Johns Hopkins School of Medicine, Baltimore, United States; 3 Department of Population Health, London School of Hygiene and Tropical Medicine, London, United Kingdom; 4 Department of Environmental Health Sciences, Columbia University, New York, United States; 5 Department of Legal Medicine, Toxicology and Physical Anthropology, Faculty of Medicine, University of Granada, Granada, Spain; 6 Department of Health, Behavior and Society, Johns Hopkins Bloomberg School of Public Health, Baltimore, United States

**Keywords:** nicotine, health implications, e-cigarettes, device characteristics, use patterns and behaviors

## Abstract

**INTRODUCTION:**

Few studies to date have characterized daily exclusive e-cigarette users, device characteristics, and use behaviors. This study describes daily e-cigarette user characteristics, and assesses the association between user behaviors and demographics.

**METHODS:**

From 2015–2017, 100 daily exclusive e-cigarette users and 50 non-users were recruited in Maryland, USA. Sociodemographic characteristics, health status, e-cigarette/tobacco use behaviors, device characteristics, and reasons for e-cigarette use were collected by interview. Chi-squared tests (categorical variables), Student’s t-test (continuous variables), and linear regressions were used to assess relationships between variables.

**RESULTS:**

Most daily exclusive e-cigarette users were men, White, former smokers, used MODs/tanks, and vaped on average 365 puffs/day (SD: 720). A third of users first vaped within 5 minutes of waking in the morning, and 56% vaped throughout the day. E-liquid consumption ranged from 5–240 mL/week (median: 32.5), with nicotine concentration 0–24 mg/mL (median: 3). E-cigarette users were more likely to report wheezing/whistling and hypertension than controls, although the finding was not statistically significant after adjustment. Less than half planned to quit vaping.

**CONCLUSIONS:**

Daily e-cigarette users between 2015–2017 most commonly vaped MOD/tank devices. Being male and of lower education was associated with higher usage. Daily users with no intention to quit may be at risk for increased exposure to emissions from e-cigarettes that include inorganic (metals) and organic (e.g. acrolein, formaldehyde) compounds with known toxic effects, particularly to the lung. Further research is needed to characterize the long-term health effects of daily e-cigarette use.

## INTRODUCTION

The use of electronic cigarettes (e-cigarettes) has significantly increased over the past decade, with rapid changes in demographic characteristics as new devices are introduced in the market. The number of current e-cigarette users in middle and high school in the US increased from 2.1 million in 2017 to 3.6 million in 2018^1^. E-cigarettes are composed of a battery, a cartridge containing e-liquid, and an atomizer, which heats and aerosolizes the e-liquid using a metal coil. E-cigarette devices have rapidly evolved in the last 10 years. First generation devices are closed, non-refillable (disposable) systems^2^ called ‘cig-a-likes’, which resemble a cigarette and consist of a cartridge (called cartomizer) that contains the e-liquid in contact with the coil, and a low-capacity rechargeable battery. The next generation devices include e-pen models (2nd generation) and tank-like systems (3rd generation), which are open, refillable, reusable systems, and were common among former smokers between 2015 and 2017^3^. Third generation open devices (called modifiable e-cigarettes or MODs) are typically larger, with a more powerful battery and adjustable voltage/wattage delivery, a refillable e-liquid reservoir, and replaceable heating coils. The fourth generation devices, called PODs, commonly used by new e-cigarette users and youth, resemble the first generation closed systems, with a low capacity battery, and a disposable ‘pod’ that contains high concentration nicotine-laden liquid in contact with a coil^4^. As of 2020, the newest generation devices include hybrid low-battery devices with refillable pods, and completely disposable devices^5^. In all generations, the heating coils used to generate the aerosol are typically made up of metal alloys. Commonly used coils include Kanthal (chromium, aluminum, iron), Nichrome (nickel and chromium), and stainless steel (nickel, chromium, carbon)^6^.

Few studies have characterized daily e-cigarette users, their devices and use behaviors, and their perceptions of e-cigarette safety. Daily e-cigarette users represent a small subgroup (19%) of the e-cigarette population compared to intermittent (29%) and occasional (51%) e-cigarette users, but they are particularly at risk of any potential long-term consequences of e-cigarette use, given the intensity of use^7^. Moreover, while nationally representative studies such as the Population Assessment of Tobacco and Health (PATH) study and the National Health Interview Survey (NHIS) have begun including questions regarding e-cigarette use, they are limited in asking questions pertaining to e-cigarette device characteristics (including voltage, power, and the type of heating coil used) and use behaviors (including the amount of e-liquid consumed per week, the number of times the heating coil is replaced per month, and the number of puffs taken per day). Describing daily use is critical in understanding the chronic exposure that could potentially result in long-term health effects.

The purpose of this study was to evaluate demographic characteristics, e-cigarette use behaviors, reasons for use, and self-reported health status of daily exclusive e-cigarette users, and to compare with matched non-users (those who neither vape e-cigarettes nor smoke combustible cigarettes). We describe e-cigarette device characteristics, vaping frequency, and e-liquid nicotine concentrations in association with user demographics among e-cigarette users in Maryland to better identify the types of users at risk and to understand the practices that may influence the potential toxicity of e-cigarettes among daily users.

## METHODS

### Study population and recruitment

E-cigarette users were recruited through advertisements and flyers posted in universities, local newspapers and online advertisement websites (City Paper, Craigslist), social media platforms (Facebook), and e-cigarette (vape) shops and conventions between December 2015 and October 2017 in Maryland, USA. Participants were residents of Maryland, aged ≥18 years, and not pregnant at the time of recruitment. The goal was to recruit 50 daily exclusive e-cigarette users during the first wave of recruitment (December 2015 to March 2016), and 50 daily exclusive e-cigarette users and 50 non-users during the second wave (March 2017 to October 2017). Exclusive e-cigarette users were defined as non-cigarette smokers or former smokers who had quit at least 6 months before enrollment and vaped daily for at least 6 weeks. Users were instructed to bring their e-cigarette devices to the study, which could either be an open or closed system. It should be noted that at the time of recruitment, none of the participants were POD users. Non-users were defined as non-cigarette smokers and non-e-cigarette users, or former cigarette smokers who quit at least 6 months prior to enrollment. To aid in the comparability, non-users were matched to e-cigarette users according to age (within 5 years), sex, and race. The study protocol was approved by the Institutional Review Board at Johns Hopkins University (Baltimore, Maryland). All participants provided written informed consent.

### Data collection

After confirming eligibility, e-cigarette user participants were asked to carry out their normal vaping routine and bring their e-cigarette device to the study visit, which took place at the Johns Hopkins Bloomberg School of Public Health in Baltimore, MD. At the time of their appointment, participants responded to an interviewer-based questionnaire addressing sociodemographic characteristics, previous tobacco use, current e-cigarette use (including e-liquid consumed/week, preferred voltage, e-liquid nicotine concentrations), overall health status, beliefs/perceptions on e-cigarette safety, and indoor vaping and smoking rules to account for potential secondary exposure. Additional questions on e-cigarette use (including the number of puffs/day, average seconds/puff, days since last coil change) were added in the second year of recruitment. Intensity of nicotine addiction was assessed by adapting the Fagerström test for nicotine dependence^8^, while sensory and respiratory symptoms were addressed using a questionnaire commonly used in studies regarding tobacco smoking and exposure to tobacco smoke^9^.

### Statistical analysis

We compared e-cigarette users and non-users by demographic characteristics, imposed house rules put in place about smoking and vaping indoors, and health characteristics using chi-squared tests for categorical variables and Student’s t-test for continuous variables. We also compared male and female e-cigarette users by primary reasons for vaping, their intention to reduce nicotine, and intention to quit vaping using chi-squared tests. Lastly, we conducted linear regression models to analyze the association of age, sex, education, race, and previous smoking status, with preferred voltage, preferred nicotine concentration, e-liquid consumed/week, puff count/day, and seconds/puff before and after adjusting for those same indicators. Statistical analyses were conducted in Stata 14 (Stata Corp, College Station, TX). The level of statistical significance was set at α=0.05.

## RESULTS

### Participant characteristics

One hundred and fifty participants (100 e-cigarette users and 50 non-users) were recruited (Table 1). Their mean age was 30.1 years (SD: 9.6), 64% were men, and 83% were White. Compared to e-cigarette users, most non-users had a higher level of education (90%) and were never smokers (90%). Eighty-nine per cent of e-cigarette users were former smokers (compared to the 10% of non-users who were former smokers); they had an earlier age to first smoke cigarettes, and smoked more cigarettes per day before quitting (mean: 17 cigarettes/day; range: 1–80 cigarettes/day) (Table 1).

**Table 1:** Participant characteristics by vaping category (N=150) ^a^

*General characteristics*	*N*	*Total*	*E-cigarette users(n=100)*	*Non-users (n=50)*	p*
**Age** (years), mean (SD)	150	30.1 (9.6)	30.3 (9.2)	29.7 (10.5)	0.70
**Gender**, %					
Male	91	64.0	67.0	60.0	0.60
Female	59	36.0	33.0	40.0	
**Education level**, %					
≤ High school	46	30.7	41.0	10.0	<0.001
>High school	104	69.3	59.0	90.0	
**Race**, %					
White	124	82.7	87.0	74.0	0.05
Non-White	26	17.3	13.0	26.0	
**Employed**, %					
Yes	99	66.0	75.0	48.0	
No	51	34.0	25.0	52.0	0.001
Student	29	19.3	9.00	40.0	
**Smoking status**					
Ever smoker, %	94	62.7	89.0	10.0	
Age (years), mean (SD)		31.2 (9.4)	30.3 (9.2)	31.6 (12.0)	<0.001
Never smoker, %	56	37.3	11.0	90.0	
Age (years), mean (SD)		28.2 (9.6)	30.3 (8.7)	29.7 (10.6)	
**Age first smoked tobacco cigarettes** ( years), mean (SD )	94	15.4 (2.9)	15.1 (2.5)	19.8 (5.7)	<0.001
**Time since quitting cigarettes** (months), mean (SD)	91	23.7 (18.2)	23.2 (18.1)	33.5 (19.8)	0.27
**Cigarettes smoked daily before quitting**, mean (SD)	92	16.3 (11.9)	16.8 (11.9)	4.5 (3.8)	0.04
**Age to first vape** (years), mean (SD)	99		28.3 (9.9)	-	-

* Comparing exclusive e-cigarette users vs non-users.

a Participants were recruited from Maryland from December 2015 – October 2017.

### E-cigarette use patterns, device characteristics, and reasons for vaping

Among e-cigarette users, the mean (SD) age at first vape was 28.3 (9.9) years (Table 1). By device type, only 2 participants used 1st generation devices while 98 users used 2nd or 3rd generation devices. More than a third (36%) of users first vaped within 15 minutes of waking in the morning, with 27% vaping within 5 minutes (Table 2). Over half of participants owned two or more devices (54%), and vaped continuously throughout the day (56%). Most users (87%) were knowledgeable about the coil, with Kanthal or some combination with Kanthal (51%), stainless steel (17%), and Nichrome (15%) being the most commonly used coils. Users’ coils were last changed at an average of 16 (SD: 19) days before coming to the study session, and replaced at an average of 3 (SD: 2) times per month. The reported mean voltage was 4.21 V (range: 2.12–12.50 V), and 85% reported periodically changing the voltage of the device. Men used a higher voltage than women, and former smokers used a lower voltage than never smokers (Table 3). According to e-liquid characteristics and use, 79% of the study population purchased their e-liquid from a vape shop, 14% online, and the remaining 7% from other sources such as making their own or receiving it from a friend. E-liquid consumption varied greatly, ranging from 5 to 240 (median: 32.5) mL/week, with women consuming less per week than men, and individuals with a higher level of education consuming less per week than those with a lower level of education. The median (range) nicotine concentration was 3.0 (0–24) mg/mL. The median (IQR) number of puffs per day was 200 (90–360) puffs, with each puff estimated to last an average of 4 (SD: 2) seconds. Older aged participants preferred higher nicotine concentrations in e-liquid and fewer puffs/day. Seconds/puff was not associated with demographic characteristics.

**Table 2:** Self-reported e-cigarette use behaviors and patterns (N=150) ^a^

*E-cigarette use behaviors*	*N*	*E-cigarette users (n=100)*
**Time to first vape** (min), %		
<5	27	27.0
6–15	9	9.0
16–30	29	29.0
31–60	24	24.0
>60	11	11.0
**Number of different devices used^a^**, %		
1	45	46.0
2	25	26.0
3	13	13.0
4	15	15.0
**Number of puffs/day^b^**, mean (SD)	50	365.1 (720.0)
**Portion of the day to vape^b^**, %		
Morning	4	8.0
Afternoon	6	12.0
Evening	12	24.0
Most of the day	28	56.0
**Average seconds/puff^b^**, mean (SD)	50	4.0 (2.0)
**E-liquid purchase location**, %		
Vape shop	77	79.0
Online	14	14.0
Other	6	6.0
**Preferred nicotine concentration** (mg/mL), median (range)	98	3 (0–24)
**E-liquid consumed per week** (mL), mean (SD)	98	53.3 (48.4)
**Device power** (W), mean (SD)		56.3 (30.8)
**Device voltage** (V), mean (SD)		4.21 (1.2)
**Change voltage**, %		
Yes	85	87.0
No	13	13.0
**How often change coil/month,** mean (SD)	96	2.5 (2.4)
**Last time of coil change** (days)^b^, mean ( SD )	50	15.9 (19.4)
**Knowledge of coil composition,** %		
Yes	87	87.0
No	13	13.0
**Type of coil used,** %		
Kanthal	44	50.6
Nichrome	13	14.9
Pure nickel	3	3.4
Stainless steel	15	17.2
Titanium	4	4.6
Combination with Kanthal	8	9.2

a Participants were recruited from Maryland from December 2015 – October 2017.

b Year 2 data only.

**Table 3:** Mean difference (95% CI) in e-cigarette use patterns by demographic characteristics analyzed using linear regression (N=150) ^a^

*Characteristic*	*Voltage (V)*			*Nicotine use (mg/mL)*			*E-liquid/week (mL)*			*Puff count/day ^b^*			*Seconds/puff ^b^*		
	*N*	*OR (95% CI)*	*AOR (95% CI)*	*N*	*OR (95% CI)*	*AOR (95% CI)*	*N*	*OR (95% CI)*	*AOR (95% CI)*	*N*	*OR (95% CI)*	*AOR (95% CI)*	*N*	*OR (95% CI)*	*AOR (95% CI)*
**Age (per year)**	92	-0.01 (-0.04, 0.02)	-0.01 (-0.03, 0.02)	98	0.24 (0.13, 0.34)	0.24 (0.12, 0.36)	98	-0.04 (-1.10, 1.02)	-0.46 (-1.55, 0.63)	50	-20.3 (-43.0, 2.39)	-25.1 (-49.9, -0.25)	50	0.01 (-0.05, 0.08)	0.02 (-0.06, 0.09)
**Gender**																	
Male	65	0.00 (Ref.)	0.00 (Ref.)	66	0.00 (Ref.)	0.00 (Ref.)	67	0.00 (Ref.)	0.00 (Ref.)	35	0.00(Ref.)	0.00(Ref.)	35	0.00(Ref.)	0.00 (Ref.)
Female	27	-0.46(-1.00, 0.07)	-0.54(-1.04, -0.03)	32	0.04(-2.24, 2.33)	0.20(-1.95, 2.34)	31	-23.5(-43.9, -3.11)	-22.7(-42.9, -2.46)	15	-103.4(-553, 347)	-132.2(-49.9, -0.25)	15	0.34(-0.88, 1.57)	0.40 (-0.92, 1.73)
**Education**																	
≤HS	38	0.00 (Ref.)	0.00 (Ref.)	41	0.00 (Ref.)	0.00 (Ref.)	41	0.00 (Ref.)	0.00 (Ref.)	19	0.00(Ref.)	0.00(Ref.)	19	0.00 (Ref.)	0.00 (Ref.)
>HS	54	-0.13 (-0.63, 0.37)	-0.12 (-0.60, 0.36)	57	-0.90 (-3.07, 1.27)	-0.02 (-2.09, 2.04)	57	-20.3 (-39.6, -0.93)	-20.4 (-39.7, -1.09)	31	202.8(-219, 625)	134.1(-301, 570)	31	-0.23 (-1.38, 0.93)	-0.25 (-1.50, 1.01)
**Race**																	
White	84	0.00 (Ref.)	0.00 (Ref.)	86	0.00 (Ref.)	0.00 (Ref.)	87	0.00 (Ref.)	0.00 (Ref.)	43	0.00(Ref.)	0.00(Ref.)	43	0.00(Ref.)	0.00 (Ref.)
Non-White	8	-0.13 (-1.00, 0.75)	-0.19 (-1.03, 0.65)	12	-2.22 (-5.47, 1.01)	-1.12 (-4.22, 1.98)	11	-25.0 (-55.5, 5.48)	-25.5 (-55.8, 4.73)	7	-245(-837, 346)	-342(-968, 284)	7	0.09(-1.53, 1.71)	0.07 (-1.74, 1.87)
**Previous smoker**																	
No	10	0.00 (Ref.)	0.00 (Ref.)	11	0.00 (Ref.)	0.00 (Ref.)	11	0.00 (Ref.)	0.00 (Ref.)	6	0.00(Ref.)	0.00(Ref.)	6	0.00 (Ref.)	0.00 (Ref.)
Yes	82	-1.28 (-2.03, -0.54)	-1.31 (-2.10, -0.53)	87	0.75 (-2.64, 4.14)	-1.09 (-4.38, 2.19)	87	0.17 (-30.7, 31.1)	-3.10 (-33.9, 27.7)	44	39.5(-597, 676)	247(-423, 919)	44	-1.04 (-1.84, 1.63)	-0.14 (-2.08, 1.79)

a Participants were recruited from Maryland from December 2015 – October 2017.

b Only year 2 data. OR: odds ratio. AOR: adjusted odds ratio, adjusted for age, gender, education level, race, and previous smoking status. HS: High school.

The primary reasons for vaping were to quit smoking cigarettes (35%), to use as a healthier alternative to cigarettes (33%), and because it is enjoyable (21%) (Table 4). The intention to reduce nicotine concentration of e-liquid was lower in women (48.9%) compared to men (70.4%). Overall, a little less than 50% of e-cigarette users reported an intention to quit vaping.

**Table 4:** Primary reasons for vaping and intention to quit (N=150) ^a^

Characteristic	N	Total%	Men%	Women%	p
**Primary reasons for vaping**	**97**				
Aid to quit smoking cigarettes	34	35.1	35.9	33.3	
Healthier than cigarettes	32	32.9	34.4	30.3	0.65
It is enjoyable	20	20.6	21.9	18.2	
Cheaper than cigarettes	5	5.2	3.1	9.1	
Other	6	6.2	4.7	9.1	
**Intention to reduce nicotine**	**99**				
Yes	60	60.6	70.4	48.9	
No	30	30.3	16.7	46.7	0.004
Don’t know	9	9.1	12.9	4.4	
**Intention to quit vaping**	99				
Yes	48	48.5	47.0	51.5	
No	27	27.3	33.3	15.2	0.11
Don’t know	24	24.2	19.7	33.3	

a Participants were recruited from Maryland from December 2015 – October 2017.

### Self-reported health status and home rules with tobacco/e-cigarette use

Regarding general health characteristics, e-cigarette users were more likely to report symptoms of wheezing and whistling in the chest (15% vs 2%, p=0.02) as well as having hypertension (22% vs 4%, p=0.007) than non-users (Supplementary file, Table S1). After running additional analyses adjusting for age, sex, and previous smoking status, this was not statistically significant. Twenty-seven of the e-cigarette users reported sensory and respiratory symptoms (sore throat, runny nose, bringing up phlegm, and coughing) occurring with e-cigarette use. While there was no difference with banning cigarette smoking inside the home between users (64%) and non-users (62%), most e-cigarette users (89%) had no rules on banning vaping indoors, compared to non-users (50%) (Supplementary file, Table S2).

## DISCUSSION

In our study sample from Maryland between 2015 and 2017, the majority of daily exclusive e-cigarette users were men, White, former smokers, and used open system devices (MODs/tanks). This is consistent with data from the nationally representative Population Assessment of Tobacco and Health (PATH) study (Waves 1 and 2), where exclusive use of e-cigarettes was more prevalent among non-Hispanic Whites compared to non-Hispanic Blacks and Hispanics^10^, and those who reported using open-system devices were more likely to report daily use compared to those who did not use this type of device^11^. Prior studies have also found ever use of e-cigarettes to be higher among men than women^12,13^. This study differs from prior e-cigarette research as it focuses on daily exclusive e-cigarette users, their behaviors and device preferences that may influence toxic exposures from daily e-cigarette use, and the differences in health characteristics and house rules of tobacco use between users and non-users.

According to the reported behaviors of e-cigarette users, close to a third of our participants first vape within 5 minutes of waking in the morning and more than half vape throughout the day, indicating a high level of nicotine dependence. Older aged individuals vaped e-liquids of higher nicotine concentrations but reported lower total puffs per day. These findings are consistent with a study of nicotine dependence and consumption among e-cigarette users mostly based in the United Kingdom, Australia, Finland, Ireland and the United States, which found that older users preferred a high nicotine-concentration and low power style of vaping^14^. With a higher level of nicotine, fewer puffs would be necessary for nicotine delivery.

E-cigarette users in our study changed their coils on average 3 times per month. No other study has reported on the frequency of coil change. This is an important behavior, as studies^15^ have found elements from coil alloys such as nickel and chromium in the aerosol that is inhaled by the user, and an increased frequency of coil change has been associated with higher metal biomarker levels^15^. The most frequently reported coil types in this study (Kanthal, stainless steel, and Nichrome) contain chromium (Cr) and/or nickel (Ni). Our group has found that the levels of these two metals in the aerosol correlate with metal levels in urine or saliva from the same participants^15^. We also found that metal levels are, in general, higher in the aerosol than in the original liquid^6^, supporting the finding that metal exposure from e-cigarette devices is likely derived, at least in part, from the heating coils. This is concerning as inhalation of nickel and chromium is known to cause airway irritation and obstruction, as well as lung, nasal, and sinus cancer^16^.

Participants reported using e-cigarettes primarily as an aid to quit smoking (35%) and as a healthier alternative to combustible cigarettes (32%). This is consistent with current established adult e-cigarette users from Wave 1 of the PATH study (2013–2014) who also reported using e-cigarettes as an alternative to cigarettes^17^. Interestingly, women in our study were less inclined to reduce their nicotine e-liquid concentrations compared to men. This is consistent with smoking cessation studies that have found that, compared to men, women have higher nicotine dependence and are less successful in quitting tobacco use^18,19^. Alternatively, it could be due to a lower nicotine flux, which is the nicotine emitted per puff-second (mg/s) that is not only determined by the e-liquid concentration used but also by the device characteristics (i.e. voltage or power settings) and the use puff topography (i.e. seconds/puff, puffs/day)^20^. Compared to men, women in our study had the same preferred mean e-liquid nicotine concentration (5.3 mg/mL). However, they vaped their devices at a lower voltage (mean difference: 0.54 V, 95% CI: -1.04, -0.03) and drew fewer puffs/day (mean difference: -132; 95% CI: -49.9, -0.25), indicating that the amount of nicotine they receive is likely lower than in men.

Overall, 48.5% of our study population intended to quit vaping altogether, which is lower than the findings from the PATH study (Wave 3: 2015–2016) where nearly two-thirds of e-cigarette users (62.4%) planned to quit e-cigarettes^21^. This PATH study sample, however, includes current e-cigarettes users who vape daily and also those who vape some days, which may account for this difference, and while 62.4% of users in the PATH study reported plans to quit, most of these users’ timeframe for quitting is a year or longer (8% plan to quit within the next 7 days, 7.7% in the next month, 13% in the next 6 months, 33% in the next year, 38% longer than that). Moreover, more than 25% of users in the PATH study reported unsuccessful e-cigarette quit attempts in the past year, indicating that quitting e-cigarette use may be a challenge, similar to quitting traditional cigarettes^21^.

Compared to non-users, e-cigarette users were more likely to report symptoms of wheezing and whistling in the chest as well as having hypertension, although after adjusting for sex, age, and former smoking status, this was not statistically significant. An assessment of Wave 2 of the PATH study also found an increased risk of wheezing and related respiratory symptoms among current e-cigarette users compared to non-users, but a lower risk when compared to current smokers or dual users^22^, which are groups we did not recruit in this study.

While our participants reported no difference in house rules regarding banning smoking cigarettes indoors, e-cigarette users were less likely to have rules in place for vaping indoors compared to non-users. This is concerning for both users and bystanders as exposure to e-cigarette aerosol is not without risks since several components have demonstrated toxicological health effects. For example, exposure to propylene glycol, a humectant in e-liquids, has been shown to decrease membrane fluidity in airway epithelia and increase mucin expression after vaping^23^. Moreover, exposure to nicotine delivered by e-cigarettes has been found to increase arterial stiffness and affect microcirculation^24^, which indicates that e-cigarettes use may be a risk factor for cardiovascular disease.

Furthermore, participants in our study reported using their devices daily, throughout the day, at a few hundred puffs per day, with behaviors that may further compound exposures. For instance, in Wave 1 of the PATH study, ‘daily’ e-cigarette users had significantly higher urinary metal levels (Pb and Sr) compared to ‘some days’ users^25^. This may be, in part, due to the increased e-liquid consumption^15^ and leaching of metals into the liquid and aerosol through contact with the coil^6^. In our study, men were more likely to vape at a higher voltage and consume more e-liquid per week than women. This higher intensity of use among men has also been reported in other studies^12,13^. There is also evidence that increasing the voltage, and thereby, power, influences reactive oxygen species (ROS) formation^26^, and shifts the particle mass distribution towards smaller particles and increases the respirable fraction of aerosol to enter ciliated airways^27^. Increasing power has also been associated with higher levels of degradation products released into the aerosol, such as aldehydes, acrolein, diacetyl, and formaldehyde^28^. Users in our study vaped at an average voltage of 4.21 V (range: 2.12–12.5) and an average of 365 puffs/day (range: 15–1000). This is concerning as Logue et al.^29^ calculated that users with a vaping regimen of 250 puffs/day using a tank device at voltages from 3.8 to 4.8 V can inhale up to 49 mg/day formaldehyde, up to 10 mg/day acrolein and up to 0.5 mg/day diacetyl, levels that exceeded US occupational limits^29^, suggesting that concentrations of these degradation products are relevant to health.

### Limitations

This study has several limitations. First, our study could be affected by selection bias due to convenience sampling. While both groups (e-cigarette users and non-users) were matched according to sex, age, and race, the majority of non-users (90%) had a higher level of education and were current students compared to e-cigarette users (59%). Second, e-cigarette use behaviors (such as e-liquid consumed/week, number of puffs/day, average seconds/puff, time to first vape after waking, etc.) are based on self-report and it is possible that participants could have displayed recall or social desirability bias. Third, as we only recruited participants aged ≥18 years, and since the use of 4th generation PODs (Juul, Suorin, etc.) rose in popularity towards the tail-end of our recruitment in 2017, we are likely missing an important population of e-cigarette users, particularly among youth of middle school and high school age.

## CONCLUSIONS

This study provides important information regarding behaviors of daily exclusive e-cigarette users. Most daily e-cigarette users were male, White, former smokers, owned an average of 2 open-system devices, and vaped an average of 365 puffs/day throughout the day. Men were more likely to vape at a higher voltage than women. Men and users with lower education consumed more e-liquid/week than their respective counterparts, suggesting a higher exposure to toxic compounds. Women expressed less desire to lower nicotine levels in their e-liquid compared to men. With daily use and no intention to quit vaping, e-cigarette users may be at risk for long-term health effects from exposures to e-cigarette by-products. Future research, in particular nationwide surveys, should document the practices of daily e-cigarette users, particularly related to the coil, power, and nicotine used in e-liquid, as the levels of both organic and inorganic compounds with known toxicological health effects are contingent on these parameters. Given the heterogeneity of e-cigarettes in the market and the ability of users to modify device characteristics, research studies looking at e-cigarette constituents and health effects should include a comprehensive characterization of e-cigarette use behaviors and device characteristics.
